# The Danger of Having All Your Eggs in One Basket—Winter Crash of the Re-Introduced Przewalski's Horses in the Mongolian Gobi

**DOI:** 10.1371/journal.pone.0028057

**Published:** 2011-12-28

**Authors:** Petra Kaczensky, Oyunsaikhan Ganbataar, Nanjid Altansukh, Namtar Enkhsaikhan, Christian Stauffer, Chris Walzer

**Affiliations:** 1 Research Institute of Wildlife Ecology, University of Veterinary Medicine, Vienna, Austria; 2 Great Gobi B Strictly Protected Area Administration, Takhin Tal, Gobi-Altai Province, Mongolia; 3 Department of Zoology, National University of Mongolia, Faculty of Biology, Ulaanbaatar, Mongolia; 4 International Takhi Group - Mongolia, Baigal Ordon, Ulaanbaatar, Mongolia; 5 International Takhi Group - Switzerland, Sihlwald, Switzerland; Université Pierre et Marie Curie, France

## Abstract

Large mammals re-introduced into harsh and unpredictable environments are vulnerable to stochastic effects, particularly in times of global climate change. The Mongolian Gobi is home to several rare large ungulates such as re-introduced Przewalski's horses (*Equus ferus przewalskii*) and Asiatic wild asses (*Equus hemionus*), but also to a millennium-old semi-nomadic livestock herding culture.

The Gobi is prone to large inter-annual environmental fluctuations, but the winter 2009/2010 was particularly severe. Millions of livestock died and the Przewalski's horse population in the Gobi crashed. We used spatially explicit livestock loss statistics, ranger survey data and GPS telemetry to provide insight into the effect of a catastrophic climate event on the two sympatric wild equid species and the livestock population in light of their different space use strategies.

Herders in and around the Great Gobi B Strictly Protected Area lost on average 67% of their livestock. Snow depth varied locally, resulting in livestock losses following an east-west gradient. Herders had few possibilities for evasion, as competition for available winter camps was high. Przewalski's horses used three different winter ranges, two in the east and one in the west. Losses averaged 60%, but differed hugely between east and west. Space use of Przewalski's horses was extremely conservative, as groups did not attempt to venture beyond their known home ranges. Asiatic wild asses seemed to have suffered few losses by shifting their range westwards.

The catastrophic winter 2009/2010 provided a textbook example for how vulnerable small and spatially confined populations are in an environment prone to environmental fluctuations and catastrophes. This highlights the need for disaster planning by local herders, multiple re-introduction sites with spatially dispersed populations for re-introduced Przewalski's horses, and a landscape-level approach beyond protected area boundaries to allow for migratory or nomadic movements in Asiatic wild asses.

## Introduction

Small populations have a high extinction risk due to demographic stochasticity, the loss of genetic variability, and the potential detrimental effect of recessive genes [1]. A small population with a restricted range resembles the proverbial “all eggs in a basket”, as it is particularly susceptible to environmental stochasticity [2]. However, many re-introduced populations of large mammals start small [3] due to logistical and financial constraints or the controversial nature of the species concerned. Large mammals re-introduced into harsh and unpredictable environments are vulnerable to stochastic effects [4], [5], particularly in times of global climate change and the associated increase in extreme weather events [6], [7].

Arid rangelands with a high level of interannual variation in precipitation are believed to follow non-equilibrium dynamics with precipitation being the main factor controlling both ungulate and vegetation dynamics [8]. The Mongolian Gobi in Central Asia constitutes a vast, largely intact and continuous stretch of non-equilibrium dry land [9] which is home to several endangered or critically endangered large migratory ungulates [10]–[12] as well as a millennium-old semi-nomadic livestock herding culture [13],[14].

Extreme weather conditions in the form of droughts followed by cold and snow rich winters (called “dzud” in Mongolia) occur at irregular intervals and have resulted in mass die-offs of livestock [15], [16]. Although periodic mass winter mortality in wild ungulates on open range has been documented elsewhere [17]–[19], [11], are frequently mentioned by local people in Mongolia and acknowledged in the scientific literature (for Asiatic wild asses *Equus hemionus* in [18], Mongolian gazelles *Procapra gutturosa* in [20], goitered gazelle *Gazella subgutturosa* in [21]), the actual impact of these climatic extremes on wild ungulate population dynamics is poorly documented, not least because of the difficulties of obtaining reliable population estimates of wild ungulates over the vast expanses of the Mongolian rangeland [22].

Przewalski's horses have been re-introduced to Mongolia since 1992 and free-ranging populations now exist in Hustai National Park (NP) in central Mongolia [23] and in the Great Gobi B Strictly Protected Area (SPA) in the Dzungarian Gobi in south-western Mongolia [24]. The initial phase of the re-introduction programme in the Dzungarian Gobi was plagued with various problems [25] and population growth could only be achieved by introducing additional captive animals. Management changes were implemented in 1999/2000, but in 2000/2001 the area was hit by a dzud winter. The population suffered a net loss of 21% and almost no foals were produced in the spring of 2001 [26]. Since 2002/03 the Przewalski's horse population finally started to show positive population growth, independent of released animals.

The positive population development in the two Mongolian re-introduction sites has resulted in the down-listing of the Przewalski's horse from “extinct in the wild” to “endangered” on the IUCN Red List of Threatened Species [27]. In December 2009 the Hustai NP population had reached 259 animals (D. Usukhjargal unpubl. data) and the Great Gobi B SPA population 138 animals or almost the “>140 horses necessary to achieve a 95% probability of survival over 100 years under the low severity level of catastrophes scenario” [26].

However, in the winter of 2009/2010 one of the worst dzud conditions ever hit Mongolia. Millions of livestock died, driving their owners into economic disaster and causing a humanitarian crisis [28], [29]. Concurrently, the Przewalski's horse population in the Great Gobi B SPA crashed, providing a textbook example of the risks faced by small and spatially confined species in unpredictable environments. By coincidence we also followed the whereabouts of 10 GPS-collared sympatric Asiatic wild asses from July 2009 until July 2010. In the following we provide insight into the effect of a catastrophic climate event on two sympatric wild equid species and the livestock population of the local semi-nomadic pastoralists in light of their different space use patterns.

## Materials and Methods

### Ethics statement

All data sets were collected within the frames of the legal requirements of Austria and Mongolia. Capture and collaring of Asiatic wild asses was conduced within a cooperation agreement between the International Takhi Group and the Mongolian Ministry of Nature, Environment and Tourism signed on 15.02.2001 and renewed on 27.01.2011.

### Study area

The Dzungarian Gobi in SW Mongolia forms a rather distinct entity of the Gobi ecosystem due to its geographic location in a basin surrounded by high mountains and by being located at the edge of the influence of the Atlantic/Mediterranean and the Asian Monsoon weather systems [30]. Almost the entire eastern and central part of the Dzungarian Gobi falls within the 9,000 km^2^ Great Gobi B SPA.

**Figure 1:**
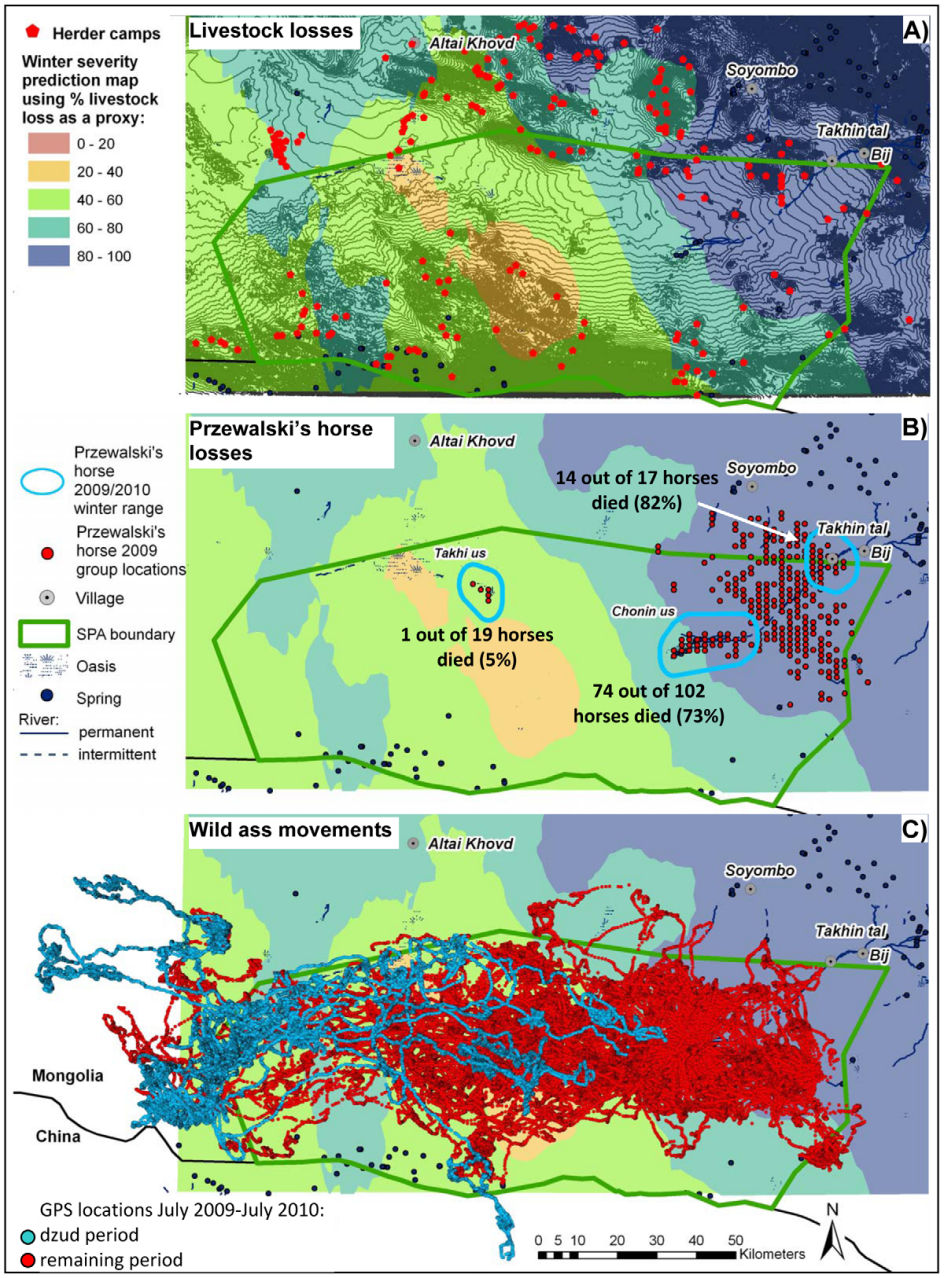
Impact of the 2009/2010 dzud winter on local nomads and two wild equid species in the Great Gobi B Strictly Protected Area in south-western Mongolia. A) Livestock loss prediction map as a proxy for winter severity based on the average % total livestock loss at 219 herder camp locations using ordinary kriging, B) Winter losses among the re-introduced Przewalski's horse population as a function of their respective winter ranges. The total distribution range in 2009 is based on group locations of 12 harem and 1–3 bachelor groups of Przewalski's horses on 129 observation days from January through December 2009. C) Movement patterns of Asiatic wild asses based on GPS positions of 10 wild asses followed from July 2009 to July 2010 (N = 355,618). Blue dots mark locations during the dzud period (N = 99,220) and red dots locations during the rest of the monitoring period.

Plains dominate the landscape of the Great Gobi B SPA in the east and rolling hills in the west. The Altai Mountains flank the park to the north, and the Takhin Shar Naruu Mountains form the international border with China (Figure 1A). Elevations range from 1,000 to 2,840 m. The climate of the Great Gobi B SPA is continental with long cold winters and short, hot summers. Average annual rainfall is 96 mm with a peak during summer. Average snow cover lasts 97 days. Both rain and snowfall can be highly variable from year to year in space and time.

Desert areas are widely dominated by Chenopodiaceae, such as Saxaul *Haloxylon ammodendron* and *Anabasis brevifolia*. The steppe areas are dominated by Asteraceae, such as *Artemisia* and *Ajania*, and Poaceae like *Stipa* and *Ptilagrostis* [31]. In locations where several springs occur, these are surrounded by intermittent swamps and form permanent oases.

The wild ungulate community of the steppe areas consists of goitered gazelle, Asiatic wild ass, and re-introduced Przewalski's horse. Starting in 1992, a total of 89 Przewalski's horses on 10 transports had been airlifted from abroad to the Takhin Tal adaptation facilities at the NE edge of the Great Gobi B SPA. Przewalski's horses live in stable harems groups, have non-exclusive home ranges of 152–826 km^2^, select for the most productive plant communities and are slow to expand their range [24]. To speed up range expansion, the last group of re-introduced Przewalski's horses, was released at the Takhi us oasis complex, about 120 km west of the established Przewalski's horse population in 2005 (Figure 1B). In 2007 three wild born stallions were flown in from Hustai NP to test the feasibility of intra-Mongolian population exchanges. Wild asses seem to live in fission–fusion groups, have non-exclusive home ranges of 4,449–6,835 km^2^ and show little preference for any particular plant community [24]. The wild ass population of the Dzungarian Gobi seems to constitute a distinct subpopulation [12], numbering several thousand individuals (P. Kaczensky and R. Ransom unpubl. data).

The Great Gobi B SPA is also used by ∼100 semi-nomadic herder families with ∼60,000 livestock. Herders show north-south seasonal movements between winter pastures along the fringes of the Dzungarian basin and alpine summer pastures in the Altai Mountains [32]. Local economy is heavily based on livestock, with cashmere generating the main income. Since the collapse of the socialist system local herders have limited access to veterinary care and largely operate without winter fodder reserves [14].

### Winter severity

We have been recording temperature on an hourly basis using a data logger (HOBO temperature logger, Hoskin Scientific Limited, Vancouver, Canada) at Takhin Tal research station since April 2003. Furthermore, O. Ganbaatar records unusual weather events in his personal research journal. No further weather stations are present anywhere in the vicinity of the Great Gobi B SPA.

### Livestock losses

To indirectly assess spatial variation in the severity of the 2009/2010 dzud conditions we obtained spatially explicit livestock loss data from the majority of local herders in and adjacent to the Great Gobi B SPA. We obtained information on livestock numbers and losses by personally interviewing local families (N = 115) and from livestock statistics collected by the local (administrative units called “bag”) governors (N = 387). We obtained winter camp coordinates either from 1∶100.000 topographic maps during our interviews or via GPS mapping in the field. Livestock numbers and losses are largely based on self-reported numbers by the local herding families.

### Przewalski's horse monitoring

We recorded births and mortalities of individual Przewalski's horses based on a *horse year* lasting from 1 May until 30 April the following year. Foals born before 1 May were manually assigned to the correct *horse year*. Population numbers for each *horse year* were calculated as the number of animals alive on 30 April, showing the net gain since the previous *horse year*. In addition, all births and mortalities, and in the past transports, were recorded by *horse year*.

Przewalski's horse groups were checked by park rangers 1–2 times a week. Rangers were able to individually identify each Przewalski's horse based on overall appearance (size, shape, coat colour, special marks) until the population numbered around 100 animals in 2007. Thereafter, they were still able to identify adult stallions and all mares, but became increasingly unsure about young stallions.

Rangers determined the location of individual Przewalski's horses and groups based on a raster map, noted group size and composition, and any peculiarities of individual horses (e.g. injuries, poor body condition, etc.). An additional 15 Przewalski's horses had been followed by satellite telemetry between 2001 and 2008 (for details see [24], [33]) and comparison between telemetry data and ranger monitoring showed that the latter is sufficient to document the broad patterns of spatial organization of the different groups and distribution range development (P. Kaczensky unpubl. data).

When rangers failed to localize individual Przewalski's horses they attempted to locate the horse's carcass by searching the area the animal was last seen in. However, during the dzud winter search efforts were hindered by deep snow and a subsequent snow melt that transformed large parts of the Gobi into mud flats. Furthermore, with the ground thawing, wildlife and domestic animal carcasses at Chonin us quickly started to sink into the many mud holes. When access finally became possible in April 2010, rangers walked the area for two full days in search of Przewalski's horse carcasses. Rangers collected various tissue samples from all carcasses encountered for histo-pathological examinations [34].

Although management of the re-introduced Przewalski's horse population has adopted a low intervention policy, emergency hay was purchased in early March 2010, and the remaining Przewalski's horses were supplemented with hay from 7 March–10 April at Chonin us and from 7–31 March at Takhin Tal and Takhi us [35]. In addition, several Przewalski's horses gained access to old hay reserves at the Takhin Tal camp from November 2009 on and received supplementary fresh hay as early as the begin of January. However, feeding was frequently disrupted by heavy snow storms that confined rangers to their homes [35].

### Wild ass monitoring

Between 2002 and 2003 we collared 7 Asiatic wild asses in the Great Gobi B SPA with Argos and GPS-Argos collars [24]. To gain more detailed insight into small scale habitat use we captured an additional 24 asses in July 2007 and July 2009 and equipped them with GPS store-on-board (SOB) collars that attempted a GPS fix every 15 minutes over a 12 month period. We retrieved 21 of the 24 collars but due to technical problems only 1 had monitored ass movements in 2007/2008 [33] while 10 had monitored wild ass movements during the dzud year 2009/2010 (collecting a total of 355,618 GPS locations, Table S1). All collars were deployed with drop-off devices (CR-2a, Telonics, Mesa, USA) programmed to release 12 months after deployment.

We attempted collar retrieval by systematically climbing high points throughout the Great Gobi B SPA and subsequently homing in on the signal from the VHF beacon of the shed units. During our search for shed GPS collars in July 2010 we also recorded all carcasses of winter-killed wild asses. Furthermore, rangers made general notes about wild ass carcasses, while searching for Przewalski's horses. Carcasses were identified as potential winter-kills, when they were fresh enough to show considerable amounts of skin with winter fur and formerly freeze-dried tissue remains.

### Data analysis

For visualization and analysis of spatial data, we used ArcMap 9.3 (ESRI, Environmental Systems Research Institute, Inc., Redlands, California, USA). We digitized rivers, springs, oases, villages and elevations from Russian 1∶100 000 topographic maps.

We created a livestock loss prediction map by using ordinary kriging in the Geostatistical Analyst function. We averaged the total livestock loss for any given winter camp if more than one family used the same location. We used the % average livestock loss of 2010 as the attribute variable to obtain the livestock loss prediction map, which we subsequently used as a spatially explicit proxy of winter severity. The proxy map was qualitatively validated by comparing it to MODIS/Terra satellite snow cover images [36] from the onset of the lasting snow cover in November 2009 and from the melt-off phase in March and April 2010 (Figure S1).

For selection of the key variables explaining death or survival of an individual Przewalski's horse between December 2009 and April 2010 we used a generalized additive model (GAM) and a generalized linear model (GLM) in R [37] with subsequent least square model averaging based on Akaike weights of all candidate models (R library glmulti [38] and MuMIn). The relative importance of each variable is expressed as the sum of the AIC weights from all models that included this variable.

## Results

### Winter severity

Dzud conditions in the Great Gobi B SPA started on 22 December 2009 and lasted until the end of March. In December 2009 and January 2010 three major snowstorms (22 December, 29 December–7 January and 17–20 January) deposited large amounts of snow, each packing down the previous layer and in places reaching accumulated snow depths of 1 m and more. In February another 5 periods of heavy snow storms occurred, each lasting for 2–3 days. From 6–8 March the last severe snow storm hit, but temperatures stayed low until mid March (Table S2, Figure S2). The period from December 2009 until March 2010 was 2.7–5.7°C colder than during the previous 7 years (Table S2).

Herders in and around the Great Gobi B SPA lost on average 67% of their entire livestock, with only camels less affected (Table 1). Most affected was the north-eastern part of the Great Gobi B SPA, where herders lost 80–100% of their livestock. Least affected were the hills in the central part of the SPA, where livestock losses were in the magnitude of 20–40%, and the areas in the west, where losses were in the magnitude of 40–60% (Figure 1A). The spatial pattern suggests that the weather largely came from the west and that the snow clouds were stopped by the high mountains forming the south-eastern tip of the Altai Mountains thereby depositing the bulk of their snow load at the north-eastern edge of the Dzungarian basin. This pattern is also supported by the timing and spatial distribution of the lasting snow cover in early November 2009 and the thawing pattern at the end of March/beginning of April 2010 (Figure S1). The majority of livestock losses occurred during the snow storms in February.

**Table 1:** Livestock losses in and around Great Gobi B Strictly Protected Area during the dzud winter 2009/2010 based on self-reported losses of 502 families.

	Livestock population		
	end Dec. 2009	N lost	% lost
Goats	80,797	54,435	0.67
Sheep	59,033	40,068	0.68
Horses	5,211	3,081	0.59
Cows/Yaks	3,377	2,066	0.61
Camels	1,049	258	0.25
***Total***	***149,467***	***99,908***	***0.67***

### Przewalski's horses

Annual population growth for the *horse years* 2002/03 until 2008/09 was positive and averaged 12% (range 1–20%; Table 2). During the *horse year* 2009/10 the population suffered a net loss of 60%. The main crash happened during the dzud period, with the population dropping from 138 Przewalski's horses in December 2009 down to 49 by the end of April 2010. Furthermore only one foal was born in 2010 (Table 2).

**Table 2:** Population development of the re-introduced Przewalski's horse population in the Great Gobi B Strictly Protected Area 1992–2010.

Horse year	Number of Przewalski's horses					Annual λ^2^
	alive by end of April	Born	dead	Winter^1^ loss	trans- ported	
1992/93	6	1	0	0	5	
1993/94	10	1	5	0	8	−1.67
1994/95	9	2	3	0	0	−1.10
1995/96	19	2	5	1	13	−1.33
1996/97	26	4	5	0	8	−1.05
1997/98	26	6	12	4	6	−1.23
1998/99	39	5	7	3	15	−1.08
1999/00	43	6	6	0	4	1.00
**2000/01** 3	**38**	**15**	**24**	**22**	**4**	**−1.21**
2001/02	35	**1**	4	0	0	−1.08
2002/03	54	13	8	2	14	1.14
2003/04	59	13	8	0	0	1.09
2004/05	86	24	9	5	12	1.25
2005/06	95	22	14	0	0	1.10
2006/07	96	33	32	3	0	1.01
2007/08	113	28	14	2	3	1.15
2008/09	124	36	25	8	0	1.10
**2009/10** 3	**49**	**28**	**103**	**89**	**0**	**−1.60**
2010/11	48	**1**	2	0	0	−1.02

1 excluding transported horses.

2 1 December until 15 April, all birth related deaths excluded.

3 Bold letters indicate horse years with a dzud winter.

The main die-off started in January (N = 16), peaked in February (N = 64) and tailed off in March (N = 6) and April (N = 3). Winter losses averaged 64% of the early December 2009 population and were most severe in the eastern winter ranges around Takhin Tal (82%) and Chonin us (73%). However, only one out of 19 animals (5%) died in the western winter range around Takhin us (Figure 1B). Most animals seem to have been lost during snow storms.

Due to the limited vehicle access of large parts of the Gobi from mid December until the middle of April, rangers could only locate the carcasses of 33 individuals. However, no living Przewalski's horses were reported from anywhere in or around the Great Gobi B SPA throughout 2010 and 2011, and thus it is safe to assume that the remaining animals also died.

Area was obviously the key factor for mortality or survival of Przewalski's horses during the dzud 2009/2010. In addition, for the 119 Przewalski's horses in the eastern winter range, age was the most likely predictor of mortality, with the youngest age classes being most affected (Table 3, Figure S3). The influence of sex and origin of the horses was less certain, but if it played a role, the effect of a zoo origin (horses born in the Gobi had a higher chance of survival than those born in a zoo) was twice as strong as the effect of sex (stallions had a lower survival probability than mares). Whether or not a mare had a foal in 2009 did not seem to have any predictive value (Table S3).

**Table 3:** Averaged model parameters of a general additive model (GAM) for survival or mortality of 119 Przewalski's horses that wintered in the eastern part of the Great Gobi B Strictly Protected Area during the dzud winter 2009/2010.

	Coefficient	z value	*P*	Relative variable importance
Intercept	−0.669	0.413	0.652	
Spline(Age)^1^	na	na	0.062^2^	0.93
Sex_Stallion	−0.855	0.472	0.070	0.68
Origin_Zoo	−1.763	0.994	0.076	0.61

1 See Figure S3 for relationship.

2 *P* value based on the full model including all 3 variables.

### Asiatic wild asses

We were able to retrieve 10 out of the 14 collars deployed in 2009. These collars had dropped off the live animals in July 2010, as they were not associated with a wild ass carcass and the data showed movements until the drop-off day. We found one collar with a non-functioning VHF unit by pure chance near a spring, suggesting that some of the 4 missing units may have had similar technical troubles. Even with the fate of 4 animals remaining unknown, a minimum of 71% of our collared asses survived the dzud winter. Furthermore, rangers did not find any ass carcasses when searching for deceased Przewalski's horses at Chonin us and registered only 1 or 2 wild ass carcasses in the Takhi us winter range. During 10 days of intensive ground search for dropped collars in July 2010 we also only came across the carcasses of 2 wild asses from the preceding winter.

The GPS data from the 10 collars revealed that wild asses had moved west during the dzud period (Figure 1C). This is a pattern we had not observed in previous years (Figure S4). Three animals even went well beyond all previous wild ass locations and one crossed the border fences between Mongolia to China and back (Figure 1C). The rangers also reported that they had not seen any wild asses in the eastern part of the park during the winter and that the very first wild ass did not arrive back in the Chonin us area until the beginning of April 2010.

## Discussion

### Herders and livestock

The dzud winter 2009/2010 was certainly the most extreme winter in Mongolia during the past 50 years. Fifteen out of Mongolia's 21 provinces were declared disaster zones and over 7.8 million livestock, 17% of the national stock, are believed to have perished [29]. The dzud disaster was caused by the combination of a very dry summer followed by a long, very cold winter with deep snow. In many places the situation was aggravated by excessive livestock stocking rates, reduced mobility and the lack of winter fodder reserves [16], [28].

Due to its geographic location, the eastern part of the Dzungarian Gobi was particularly heavily hit. In and around the Great Gobi B SPA stocking rates are moderate, although numbers were on the rise (from ∼60,000 in 2001, see [32], to ∼75,000 by the end of 2009). While there is little indication for pasture degeneration so far, there is evidence for competition for good winter camps (e.g. suboptimal campsites being used, and camps being burnt down as acts of sabotage), which constitute a key resource for local herders [13].

Competition for winter camps allows little spatial flexibility and when combined with a lack of infrastructure and supporting services families basically had to stay put, even when the conditions were bad. Furthermore, during the 2009/2010 dzud winter deep snow arrived so quickly that people were unable to move, even if they had an alternative place to go to. Several families in the eastern part of the Great Gobi B SPA got trapped at fall camps, which are normally only used for a few weeks after leaving the summer pastures and before settling into the winter camps. These families had extended their presence at the temporary fall camps unusually long because of the rather poor condition of their winter pastures caused by the preceding summer's drought.

### Przewalski's horses

The weather conditions during the dzud 2009/2010 resulted in the north-eastern part of the Dzungarian basin receiving large amounts of snow. This area also happened to be the winter range of the majority of the re-introduced Przewalski's horses, resulting in a major population crash. A modelling exercise had previously identified natural catastrophes as having the greatest influence on the extinction risk of the small Przewalski's horse population in Takhin Tal [26]. However, the frequency, spatial extent and severity of such unusual weather events are difficult to predict, making long term model predictions of population growth of small populations in harsh environments even more unreliable than under “normal environmental stochasticity” [4].

Provision of hay after the main die-off may have helped to reduce late winter mortality at Chonin us and Takhi us, explaining the slightly lower losses among Przewalski's horses as compared to livestock. However, around Takhin Tal access to hay from early winter on did not prevent massive losses, possibly because feeding was impossible during snow storms [35]. Consequently, intervention possibilities for free-ranging animals during natural disasters of the magnitude of the 2009/2010 dzud seem very limited.

On the regional scale the damage to Przewalski's horse recovery in Mongolia was somewhat dampened by the fact that the winter range of the third Przewalski's group in the Great Gobi B SPA was located in an area less affected by the dzud, and on a national scale by the fact that winter losses at Hustai NP were much lower and only amounted to 10% of the early December population (D. Usukhjargal unpubl. data). Close cooperation between the two re-introduction sites already resulted in a transport of horses from Hustai NP to the Great Gobi B SPA and negotiations for further transports to speed up population recovery are ongoing. Furthermore, there are plans to transport Przewalski's horses from a breeding facility in adjacent China to Mongolia.

As during the dzud 2000/2001, the youngest age classes suffered the highest mortality [26]. What came as a surprise though was that zoo born Przewalski's horses, despite having lived in the Gobi for multiple years, may still have a lower survival probability than those born in the Gobi. It also appears that mares may be less susceptible to succumbing to dzud conditions than stallions. Histo-pathological examination of samples collected from 33 Przewalski's horse carcasses did not suggest that disease played a major role in the dzud 2009/2010 die-off (A. Kübber-Heiss, unpubl. data), as had been the case during the dzud 2000/2001 [34]. Our findings suggest that with the increasing proportion of Gobi born Przewalski's horses the re-introduced population may become more robust in facing future dzud conditions, although we have yet to understand the underlying adaptive mechanisms.

Although, contrary to livestock, Przewalski's horses were not constrained to any particular place by the choice of a herder, they nevertheless stayed put in the area most affected by the dzud. Access to hay from early winter on may have been a reason for the 17 Przewalski's horses around Takhin Tal to remain. However, the 102 horses at Chonin us did not receive hay until after the main die-off had already happened. The area most heavily impacted included the horses' winter and summer range. The re-introduced Przewalski's horses seem very conservative in their range use, having comparatively small home ranges, clear habitat preferences, and showing only a slow tendency for range expansion [24]. Przewalski's horse groups in the north-eastern part of the SPA overlap and interact, but there seems to be no contact to the group at Takhi us (O. Ganbaatar unpubl. data). Consequently, the re-introduced population still has a rather limited spatial knowledge, and venturing beyond the known range during extreme conditions would be somewhat of a risky strategy. Whether autochthonous Przewalski's horses were more mobile than the present-day re-introduced animals is unknown. From other species we know that sedentary and migratory animals or subpopulations can coexist within the same species and habitat [39]. Thus it is possible that during captive breeding either the behavioural tradition or the genetic component for exploratory movements was lost. However, the severe effect of this localized catastrophic event was largely due to the small size and limited range of the present day Przewalski's population. A large and continuous population would be able to counteract local population lows or extinctions via re-colonization.

### Asiatic wild asses

Although we have no means of quantifying the impact of the dzud 2009/2010 on the Asiatic wild ass population, evidence suggests that mortality was low. GPS tracking data and ranger observations show that wild asses moved away from the most severely affected areas in the north-eastern part of the Dzungarian Gobi, a pattern we had not observed in previous years [12], [24], (also see Figure S4). Contrary to sympatric Przewalski's horses, wild asses have large home ranges, show little dependence on a particular habitat type and seem to live in fission-fusion groups [24]. Due to the different scale of habitat use, winter severity within the asses' home range was patchy, rather than uniform as was the case within the much smaller Przewalski's horse home ranges, or punctual like for the fixed winter camps of local herders and their livestock. The lack of a distinct spatial substructure within the wild ass population [12] likely facilitates information transfer between individuals and subgroups [40] and may allow individuals access to information well beyond their individual home range [41], making exploratory long distance movements during extreme conditions less risky.

### Management Recommendations

The dzud winter 2009/2010 is a text book example for how vulnerable small and spatially confined populations are in an environment prone to fluctuations and catastrophes. Losses of this magnitude are difficult to predict or model in any reasonable framework and will in any case remain probabilities. As long as populations remain small and spatially confined, success is not guaranteed, necessitating a long term conservation commitment.

The difference in how severely the Przewalski's horse population was affected, on a local as well as a national scale, highlights the advantage of distributing your “eggs in more than one basket”. Consequently, the national strategy for Przewalski's horse conservation in Mongolia should continue to aim for multiple re-introduction sites with spatially dispersed populations and close cooperation among projects on a national as well as an international scale.

Wild asses were obviously able to avoid the worst of the dzud winter by moving west, up to 50 km beyond the Great Gobi B SPA boundary. These long distance movements and range shifts highlight again how vulnerable migratory or nomadic ungulates are to fragmentation and how important it is to manage them on a landscape-level, including multi-use areas outside of protected areas.

The spatial flexibility of local herders is restricted by the limited availability of suitable winter camps and further complicated by administrative boundaries and a lack of cooperation beyond the extended family. Consequently, outrunning a dzud disaster is hardly an option and people will need to prepare for dzud events by means of banking during good years, improved husbandry and control of stocking rates and diversification of their means of income. Certainly, the herding sector will not be able to provide a livelihood for a growing population in the future.

## Supporting Information

Figure S1 **Snow cover dynamics from the first snowfalls in November 2009 to snow melt in April 2010, over- imposed with high loss areas from the % livestock loss prediction map.** Generally, high loss areas correspond with areas that received snow early and where snow stayed long. Snow depth can be indirectly inferred from snow melt patterns. Quantitative analyses were hindered by 1) the inability to remotely measure snow depth, and 2) the high percentage of satellite images with total or partial cloud cover (e.g. images top left & images in the middle), resulting in large no-data zones.
(DOC)

Figure S2 **Snow conditions in and around Takhin Tal in February and March 2010.**
(DOC)

Figure S3 **Probability of mortality for the 119 Przewalski's horses that wintered in the east part of the Great Gobi B SPA during the dzud winter 2009/10 based on age.** The solid line shows the value predicted by the general additive model (GAM) based on a spline with 8 knots. The dashed lines are the 95% credibility intervals.
(DOC)

Figure S4 **GPS positions of 8 wild asses between July 2002 and July 2009, years with no dzud winters.** No avoidance of the eastern part of the park, as in 2009/10, is seen. For detailed description of data collection and monitoring period see [24] and [33].
(DOC)

Table S1 **GPS data from 10 Asiatic wild asses monitored from July 2009 until July 2010.**
(DOC)

Table S2 **Temperatures based on hourly measurements at Takhin Tal research station at the NE edge of the Great Gobi B SPA in SW Mongolia.** A) Average monthly temperatures from April 2003 through August 2010. B) Daily mean temperature from November 2009 through April 2010.
(DOC)

Table S3 **Averaged model parameters of a general linear model (GLM) for survival or mortality of 39 adult Przewalski's horse mares (age≥4 years) that wintered in the eastern part of the Great Gobi B SPA during the dzud winter 2009/10.**
(DOC)
