# Supplementary material for: The Danger of Having All Your Eggs in One Basket—Winter Crash of the Re-Introduced Przewalski's Horses in the Mongolian Gobi
Source: PLoS One. 2011 Dec 28;6(12):e28057. doi: 10.1371/journal.pone.0028057 (PMC3247207; doi:10.1371/journal.pone.0028057)
Supplement: Figure S1 — Snow cover dynamics from the first snowfalls in November 2009 to snow melt in April 2010, over- imposed with high loss areas from the % livestock loss prediction map. Generally, high loss areas correspond with areas that received snow early and where snow stayed long. Snow depth can be indirectly inferred from snow melt patterns. Quantitative analyses were hindered by 1) the inability to remotely measure snow depth, and 2) the high percentage of satellite images with total or partial cloud cover (e.g. images top left & images in the middle), resulting in large no-data zones. (DOC) [file pone.0028057.s001.doc]

**Figure S1.**


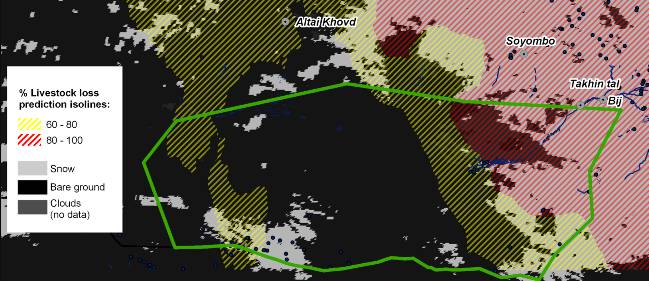

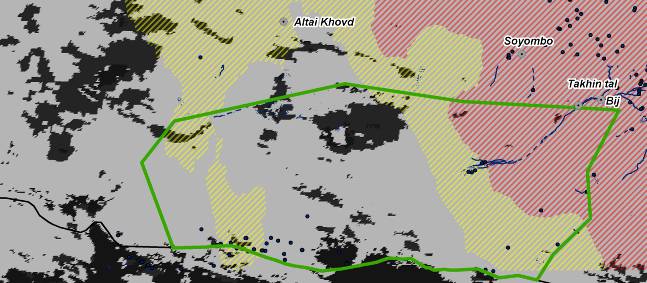

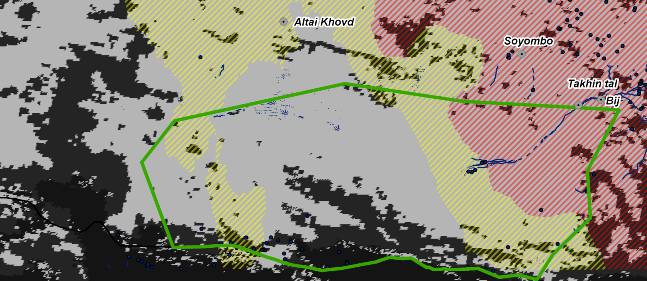

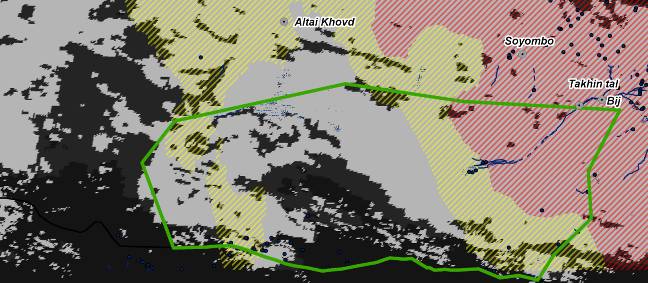

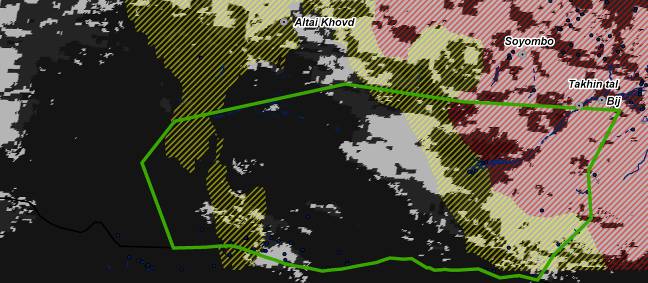

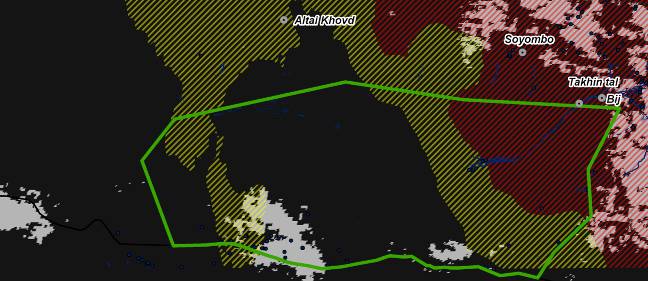


01.11.-09.11.2009

17.11.-25.11.2009

06.03.-14.03.2010

22.03.-30.03.2010

07.04.-15.04.2010

23.04.-01.05.2010

*Eight-day snow cover layers were obtained as 500m grids from the National Snow and Ice Data Center (NSID), for further details see [36]. Data available from:* [*http://nsidc.org/data/modis/order_data.html*](http://nsidc.org/data/modis/order_data.html)*.*
