# Supplementary figures and images for: The Danger of Having All Your Eggs in One Basket—Winter Crash of the Re-Introduced Przewalski's Horses in the Mongolian Gobi
Source: PLoS One. 2011 Dec 28;6(12):e28057. doi: 10.1371/journal.pone.0028057 (PMC3247207; doi:10.1371/journal.pone.0028057)

**Figure S2.**


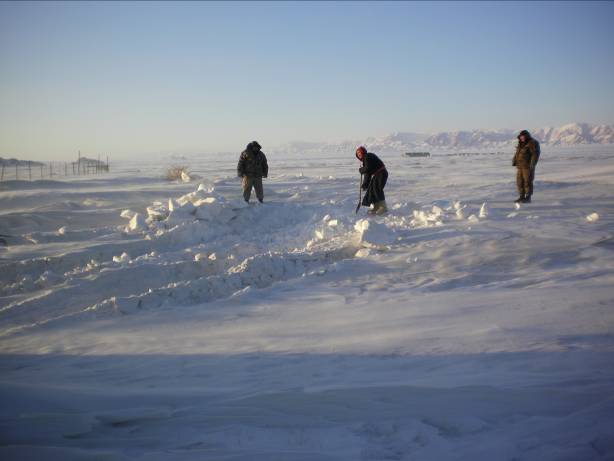

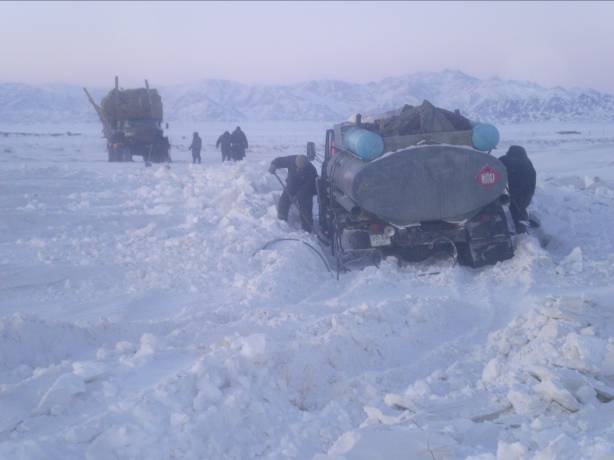

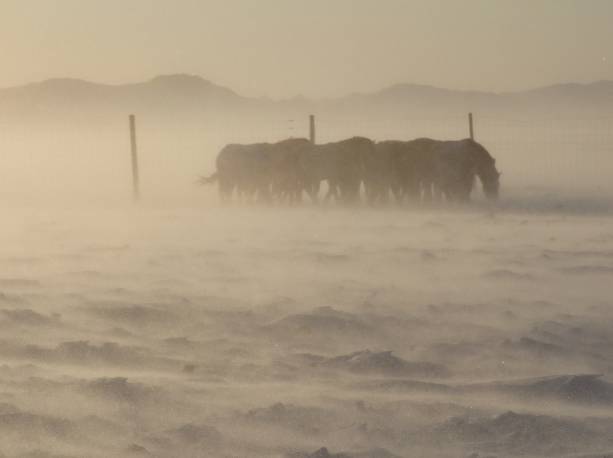

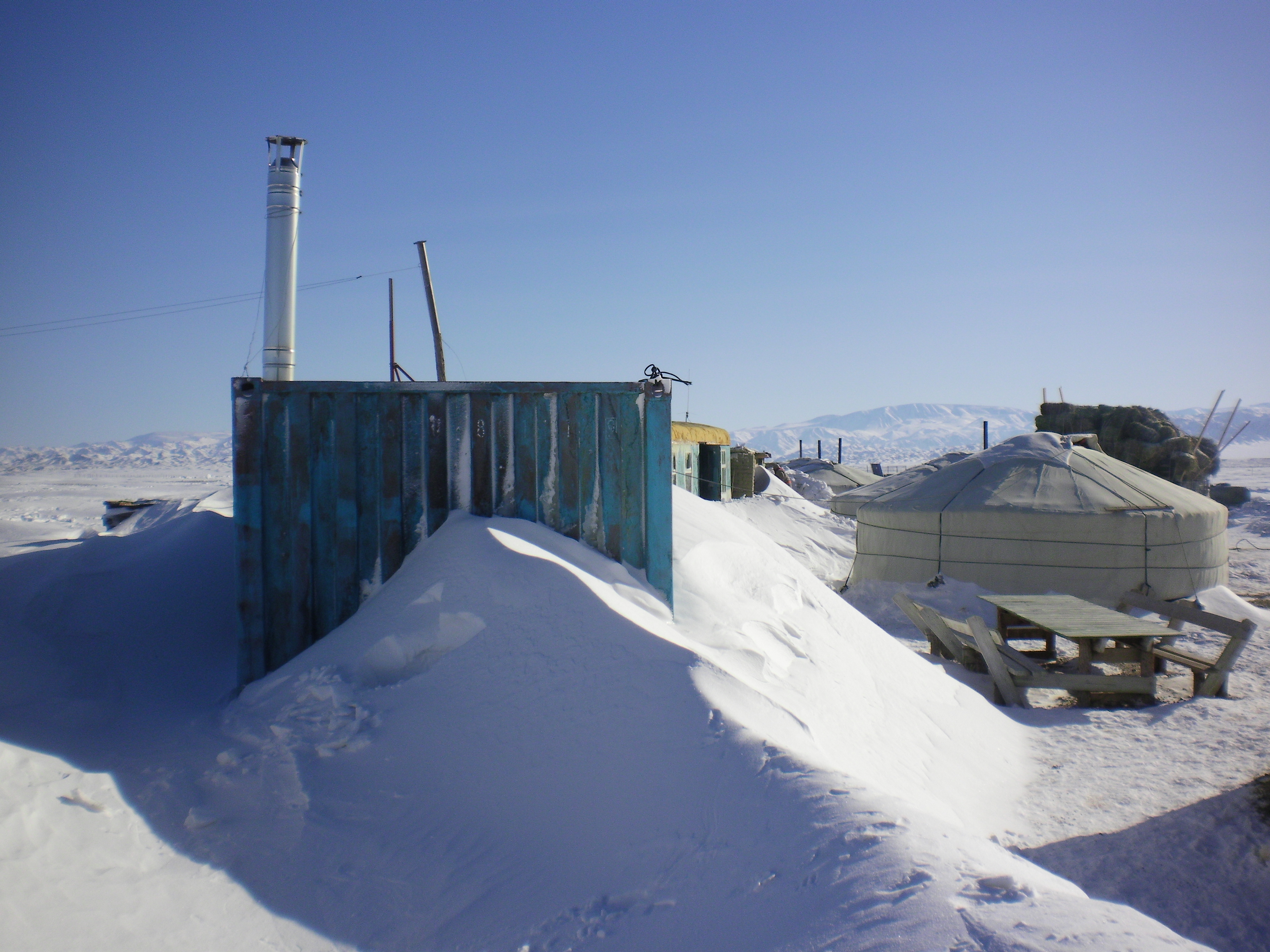


*Photos: O. Ganbaatar / N. Altansukh / G. Nisekhuu.*

Supplement: Figure S2 — Snow conditions in and around Takhin Tal in February and March 2010. (DOC) [file pone.0028057.s002.doc]

**Figure S3.**


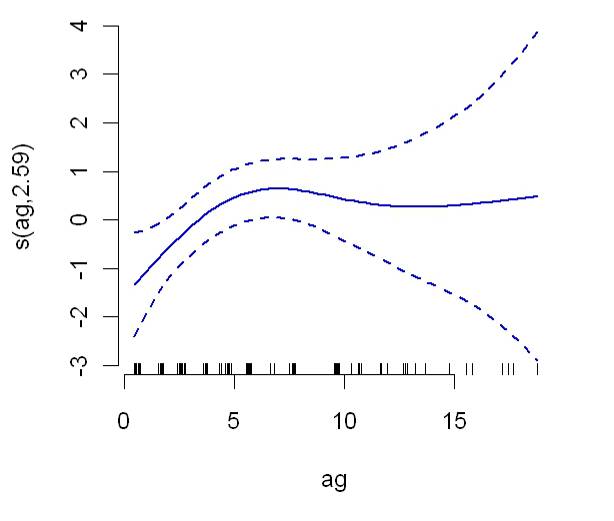


Age (years)

Supplement: Figure S3 — Probability of mortality for the 119 Przewalski's horses that wintered in the east part of the Great Gobi B SPA during the dzud winter 2009/10 based on age. The solid line shows the value predicted by the general additive model (GAM) based on a spline with 8 knots. The dashed lines are the 95% credibility intervals. (DOC) [file pone.0028057.s003.doc]

**Figure S4.**


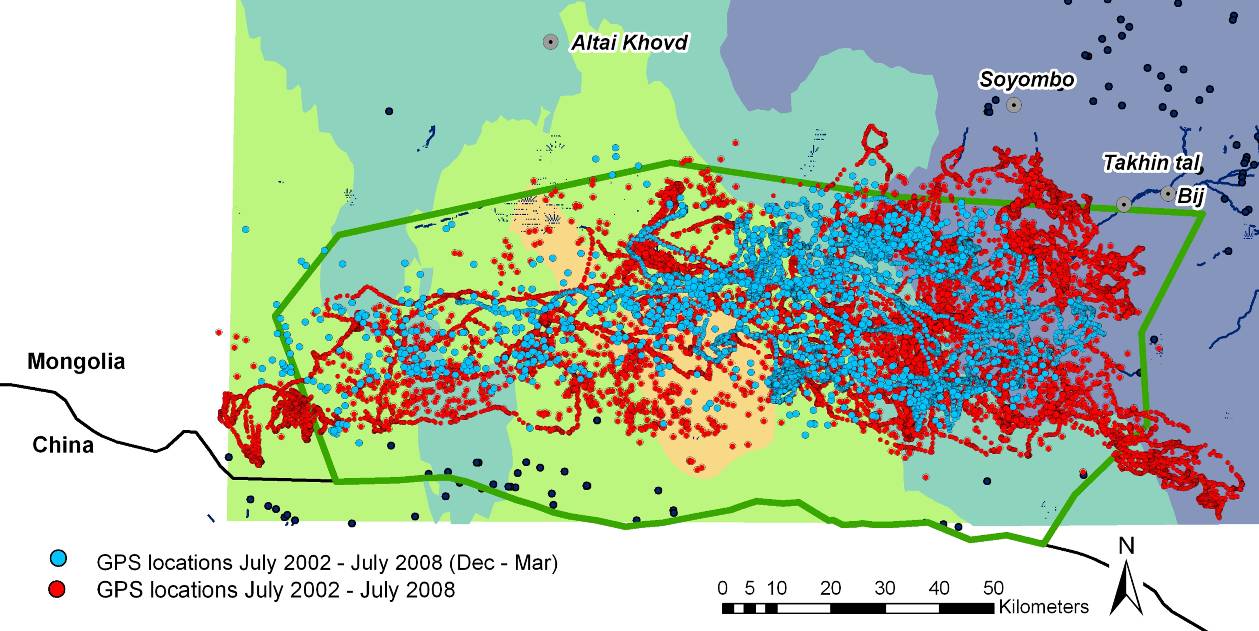


GPS locations July 2002-July 2009:

209/2010 dzud period

remaining period

Supplement: Figure S4 — GPS positions of 8 wild asses between July 2002 and July 2009, years with no dzud winters. No avoidance of the eastern part of the park, as in 2009/10, is seen. For detailed description of data collection and monitoring period see [24] and [33]. (DOC) [file pone.0028057.s004.doc]
