# Supplementary material for: The Danger of Having All Your Eggs in One Basket—Winter Crash of the Re-Introduced Przewalski's Horses in the Mongolian Gobi
Source: PLoS One. 2011 Dec 28;6(12):e28057. doi: 10.1371/journal.pone.0028057 (PMC3247207; doi:10.1371/journal.pone.0028057)
Supplement: Table S1 — GPS data from 10 Asiatic wild asses monitored from July 2009 until July 2010. (DOC) [file pone.0028057.s005.doc]

**Table S1.**

| **#** | **Capture date** | **Sex** | **Age** | **GPS locations** | | **N Monitoring days** |
| --- | --- | --- | --- | --- | --- | --- |
|  |  |  |  | **all** | **during dzud** |  |
| 1 | 21.07.09 | mare | 8 | 21,774 | 13,329 | 169 |
| 2 | 22.07.09 | mare | 15 | 24,977 | 10,959 | 235 |
| 3 | 20.07.09 | mare | 5-6 | 40,643 | 11,308 | 366 |
| 4 | 20.07.09 | stallion | 3 | 40,207 | 11,486 | 366 |
| 5 | 23.07.09 | mare | 8-9 | 31,232 | 13,006 | 259 |
| 6 | 20.07.09 | stallion | 5-6 | 14,179 | 0 | 132 |
| 7 | 24.07.09 | stallion | 12 | 37,387 | 10,356 | 362 |
| 6441 | 22.07.09 | stallion | 15 | 34,815 | 9,596 | 364 |
| 6446^1^ | 21.07.09 | mare | 15 | 34,908 | 9,598 | 365 |
| 7376 | 23.07.09 | mare | 2-3 | 34,673 | 9,582 | 363 |
| 4958 | 22.07.09 | stallion | 12 | not retrieved | | |
| 6284 | 24.07.09 | mare | 9-10 |  |  |  |
| 6447 | 21.07.09 | stallion | 7 |  |  |  |
| 7020 | 21.07.09 | mare | 4 |  |  |  |

^1^Collar found by pure chance next to a spring. The VHF unit had ceased working.

^2^Collars 1-7 were FIWI GPS-SOB units and again suffered from some software irregularities explaining the differences in the number of locations and monitoring days.
