# Supplementary material for: The Danger of Having All Your Eggs in One Basket—Winter Crash of the Re-Introduced Przewalski's Horses in the Mongolian Gobi
Source: PLoS One. 2011 Dec 28;6(12):e28057. doi: 10.1371/journal.pone.0028057 (PMC3247207; doi:10.1371/journal.pone.0028057)
Supplement: Table S2 — Temperatures based on hourly measurements at Takhin Tal research station at the NE edge of the Great Gobi B SPA in SW Mongolia. A) Average monthly temperatures from April 2003 through August 2010. B) Daily mean temperature from November 2009 through April 2010. (DOC) [file pone.0028057.s006.doc]

**Table S2.**

*A)*

| **Month** | **Year** | | | | | | |  |
| --- | --- | --- | --- | --- | --- | --- | --- | --- |
|  | **2003** | **2004** | **2005** | **2006** | **2007** | **2008** | **2009** | **2010** |
| 1 | - | -18 | -21 | -20 | -18 | -21 | -18 | -19 |
| 2 | - | -16 | -21 | -16 | -12 | -15 | -18 | -20 |
| 3 | - | -11 | -4 | -7 | -6 | -1 | -6 | -14 |
| 4 | 14 | 6 | 5 | 3 | 6 | 4 | 8 | -3 |
| 5 | 10 | 12 | 11 | 10 | 12 | 13 | 11 | 9 |
| 6 | 19 | 17 | 16 | 17 | 17 | 19 | 15 | 18 |
| 7 | 17 | 20 | 20 | 19 | 20 | 20 | 19 | 20 |
| 8 | 15 | 16 | 17 | 18 | 17 | 18 | 16 | 15 |
| 9 | 11 | 9 | 11 | 11 | 12 | 11 | 11 | - |
| 10 | 2 | 1 | 2 | 3 | 0 | 3 | 3 | - |
| 11 | -12 | -10 | -9 | -5 | -6 | -9 | -13 | - |
| 12 | -19 | -15 | -18 | -17 | -14 | -19 | -21 | - |
| mean  Dec-Mar | - | -15.74 | -15.22 | -15.25 | -12.94 | -12.73 | -15.12 | -18.44 |

*B)*
