# Supplementary material for: The Danger of Having All Your Eggs in One Basket—Winter Crash of the Re-Introduced Przewalski's Horses in the Mongolian Gobi
Source: PLoS One. 2011 Dec 28;6(12):e28057. doi: 10.1371/journal.pone.0028057 (PMC3247207; doi:10.1371/journal.pone.0028057)
Supplement: Table S3 — Averaged model parameters of a general linear model (GLM) for survival or mortality of 39 adult Przewalski's horse mares (age≥4 years) that wintered in the eastern part of the Great Gobi B SPA during the dzud winter 2009/10. (DOC) [file pone.0028057.s007.doc]

**Table S3.**

|  | **Coefficient** | **z value** | ***P*** |  | **Relative variable importance** |
| --- | --- | --- | --- | --- | --- |
| Intercept | 0.428 | 0.451 | 0.652 |  |  |
| Age^1^ | -0.090 | 0.593 | 0.553 |  | 0.33 |
| Foal_yes | -0.875 | 1.050 | 0.294 |  | 0.36 |
| Origin_Zoo | -1.893 | 2.057 | 0.040 | * | 0.79 |

^1^Age was not included as a spline, like for the full model (Table 3 and Figure S3), because adult mortality did seem to be more or less constant with a high uncertainty for very old horses.

* variable significant on the *P* < 0.05 level
